# Quantifying the correlation between spatially defined oxygen gradients and cell fate in an engineered three-dimensional culture model

**DOI:** 10.1098/rsif.2014.0501

**Published:** 2014-09-06

**Authors:** Amir G. Ardakani, Umber Cheema, Robert A. Brown, Rebecca J. Shipley

**Affiliations:** 1 University College London, Tissue Repair and Engineering Centre, Institute for Orthopaedics and Musculoskeletal Sciences, Stanmore Campus, London HA7 4LP, UK; 2 University College London, Biomechanical Engineering Group, Department of Mechanical Engineering, London WC1E 7JE, UK

**Keywords:** cell culture, biocompatibility, collagen, tissue engineering

## Abstract

A challenge in three-dimensional tissue culture remains the lack of quantitative information linking nutrient delivery and cellular distribution. Both *in vivo* and *in vitro*, oxygen is delivered by diffusion from its source (blood vessel or the construct margins). The oxygen level at a defined distance from its source depends critically on the balance of diffusion and cellular metabolism. Cells may respond to this oxygen environment through proliferation, death and chemotaxis, resulting in spatially resolved gradients in cellular density. This study extracts novel spatially resolved and simultaneous data on tissue oxygenation, cellular proliferation, viability and chemotaxis in three-dimensional spiralled, cellular collagen constructs. Oxygen concentration gradients drove preferential cellular proliferation rates and viability in the higher oxygen zones and induced chemotaxis along the spiral of the collagen construct; an oxygen gradient of 1.03 mmHg mm^−1^ in the spiral direction induced a mean migratory speed of 1015 μm day^−1^. Although this movement was modest, it was effective in balancing the system to a stable cell density distribution, and provided insights into the natural cell mechanism for adapting cell number and activity to a prevailing oxygen regime.

## 1. Introduction

A key requirement of any tissue-engineered construct is the ability to mimic *in vivo* cellular function in an *in vitro* construct. This necessitates a three-dimensional culture environment in which both the mechanical and chemical environment of the cells can be controlled and cellular function (e.g. gene expression and cellular morphology) tested [1–3]. Biocompatibility, integration once implanted *in vivo*, material support of the cell population, mechanical integrity, a defined chemical environment and the ability to mimic features of native tissue architecture are all of fundamental importance. These present a significant challenge that may be addressed by removing cells from their native tissue and culturing them in hydrogels loaded with native extracellular matrix components [4–6]. A range of such natural biomaterials have been explored, including collagen [7], gelatin [8] and fibrin [9]; collagen is the most prevalent choice, as it is the dominant protein component of connective tissues and comprises 25–35% of total body protein content. These gels overcome challenges of biocompatibility and biodegradability associated with their synthetic counterparts; however, they have traditionally been limited by their mechanical strength [10].

Plastic compression of cell-laden gels addresses this mechanical deficiency by producing dense cell-containing collagen materials with tissue-like collagen fibril density and organization [1,11–13]. In plastic compression, excess fluid is removed in a controlled manner from the initial collagen gel to produce collagen densities of around 12% [11], with corresponding increase in mechanical strength. This cell enmeshing effect avoids complex problems associated with cell-dependent matrix production or modification or synthetic scaffold replacement. Further, the collagen densities generated are typical of native collagen type I tissues, providing a more accurate model of three-dimensional tissue [1,11]. The compressed matrices have good biomimetic and functional mechanical properties with regards to compliance and strength, alongside both nano- and microscale structure that supports good cell viability and physiological patterns of remodelling behaviour [1,14].

Mass transport of nutrients (e.g. oxygen, glucose), waste materials (e.g. lactate) and proteins (e.g. growth factors) is essential to sustain an evolving, functional cell population. Oxygen is usually the limiting nutrient (this is a consequence if its weaker solubility in water compared with, for example, glucose); hypoxia also provides the chemical cue for vascularization *in vivo*. As a consequence, oxygen has been the focus for extensive transport and vascularization studies [15–20]. In healthy tissues, oxygen is supplied to cells by diffusion from the nearest capillary; the oxygen environment experienced by a particular cell is a function of the local oxygen tension in the perfusing capillary network, the distance for oxygen diffusion between the cell and the nearest capillary and the metabolic uptake of oxygen by the cell population. As oxygen diffuses from a capillary into a tissue, it is metabolized by cells in proportion to the underlying cellular density; therefore, the distance before hypoxia is a complex tension between the rates of diffusion versus metabolism. Consequently, tissue oxygen environment is not only cell-type-specific, but also spatially varied. Such gradients in oxygen tension are a feature of the normal physiological scenario, and need to be mimicked in an *in vitro* set-up. Surprisingly, in view of much popular opinion, the collagen material as a ‘diffusion barrier’ is actually a minor factor in this balance [1,21].

Chemical gradients are tightly correlated with cellular density through proliferation, death and chemotaxis. Although a higher oxygen environment, in general, drives a faster rate of proliferation (e.g. fibroblasts [22]), a low oxygen environment may induce death (either through necrosis or apoptosis); the effects on differentiation or synthesis are less clear. In the absence of other effects, proliferation and death would therefore give rise to a cell density gradient correlated with the underlying oxygen gradient; cell density would be highest in well-oxygenated regions, and conversely lowest in oxygen-deprived zones. This is complicated further for cell types that exhibit motility through chemotactic behaviours. Chemotaxis is the directed migration of cells up gradients of soluble molecules or chemoattractants. It is of fundamental importance in cancer metastasis [23], embryogenesis [24] and wound healing [25]; although such chemotactic behaviours are commonly described (e.g. fibroblasts [26]), thorough quantification of the relationship between chemotaxis and underlying chemoattractant gradients remains rare (notable exceptions include those in neutrophils and leucocytes [27–30]). The aim of this study was to quantify the relationship between oxygen gradients and cell fate (incorporating proliferation, death and chemotaxis) for three-dimensional plastic-compressed collagen constructs.

**Figure 1.**
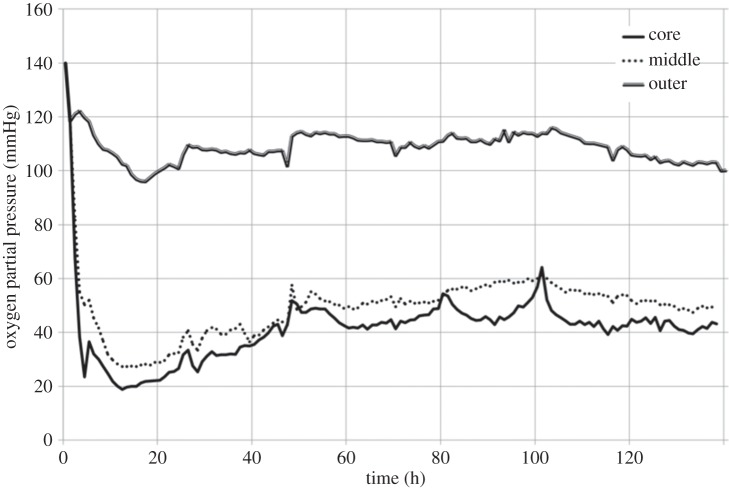
Continuous measurement of oxygen partial pressure in the outer, middle and core regions of the plastic-compressed collagen construct. Results previously reported [1]. Construct seeded with 23.2 million HDF cells ml^−1^. Significant oxygen gradients were quantified moving from the outer region inwards towards the construct core.

In previous studies, the current authors have quantified oxygen gradients in a simple and well-established plastic-compressed collagen type I model [1,31]. A sheet of cell-laden compressed collagen matrix was spiralled and cultured in a vessel of nutrient-rich medium, replenished at regular time intervals; oxygen (among other nutrients) is supplied to cells embedded throughout the matrix via passive diffusion from the outer surface of the spiral. The oxygen status throughout such scaffolds has been assessed using fibre-optic probes (Oxford Optronix, Oxford, UK) positioned in the outer, middle and core regions of the spiralled constructs (figure 1). Ambient oxygen partial pressures in the culture medium remained at approximately 140 mmHg throughout the experiments (7.6 mmH corresponds to 1% oxygen). For both human dermal fibroblasts (HDFs) and human bone mesenchymal stem cells (HBMSCs) at cell densities in the range 0.5–2 million cells per construct and a collagen density of 11.6%, the oxygen gradient established as a consequence of cellular metabolism resulted in typical jumps in the oxygen partial pressure from 100 to 40 mmHg moving from the outer region to the core [1]. These jumps in oxygen partial pressure are consistent with physiological values [1,31]. This study analyses these data alongside new spatially resolved data on HDF proliferation, viability and chemotaxis acquired through analysis of the outer, middle and core of collagen type I spiralled constructs over a 10-day time period. Preferential proliferation and viability are demonstrated and quantified in the highest oxygen zones (i.e. the outer region); the route and extent of chemotaxis from the core to the middle/outer regions over the 10-day period are also quantified. This represents the first time such detailed correlations have been possible under such biomimetic, three-dimensional-controlled conditions.

## 2. Material and methods

### 2.1. Cell culture and expansion

HDFs were obtained from neonatal foreskins (obtained freshly from the operating theatre, with full ethical approval, following surgery for circumcision and then cryopreserved) as described by previous work [1,21,31]. HDFs were used with passage numbers 6 and 8 in all experiments. Cells were cultured and maintained in high glucose (4500 mg ml^−1^) Dulbecco's modified Eagle's medium (Gibco, Paisley, UK), supplemented with 10% fetal calf serum (First Link, West Midlands, UK), 1000 U ml^−1^ penicillin, 100 μg ml^−1^ streptomycin and 2 mM glutamine (all from Gibco Chemicals, UK). In order to remove the monolayer of cells culturing on the surface of the flask, 225 ml flasks containing cells were washed twice with 0.1 M phosphate-buffered solution (PBS) followed by incubation with trypsin (0.5% in 5 mM EDTA) for 5 min at 37°C. Once cells were separated from the surface of the flask culture medium, double the amount of trypsin was added; the mixture was put into a universal tube, centrifuged at 2000 r.p.m. for 5 min and cell numbers counted.

### 2.2. Three-dimensional plastic compression and collagen scaffold preparation

**Figure 2.**
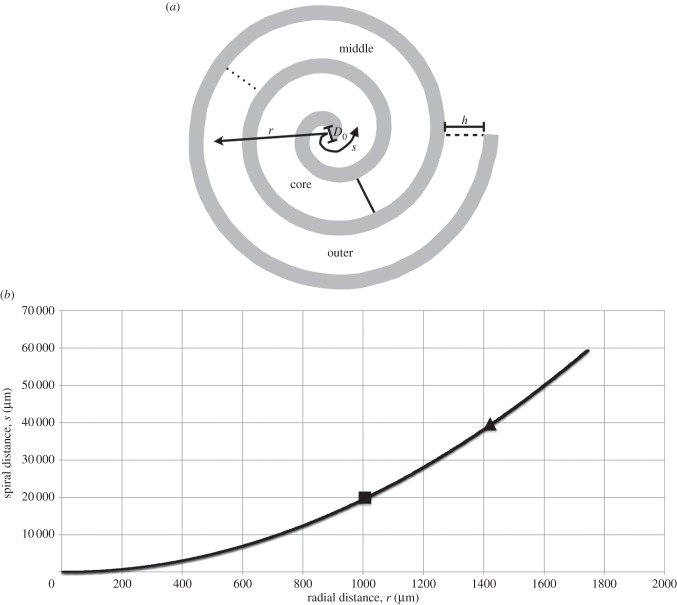
Experimental set-up and definition of radial versus spiral distances. (*a*) A schematic of a spiralled construct depicting the characterizing parameters together with spiral (*s*) versus radial (*r*) distance definitions. The collagen construct is coloured white, with surrounding fluid in grey; spiral distances are measured from the origin following the centreline of the collagen spiral, whereas radial distances are measured radially outwards. The spiral geometry is defined by its inner diameter *D*_0_, the number of turns *N* (=3 in this schematic) and the thickness of the collagen sheet *h*. Example core, middle and outer regions are annotated; these are defined in the spiral coordinate system once the spiral is unrolled and divided evenly. (*b*) The relationship between spiral and radial distances for the spiral parameters in this study (*D*_0_ = 80 μm, *N* = 9 and *h* = 80 μm). Boundaries between the core/middle and middle/outer regions denoted with a square and triangle, respectively.

Cell-seeded collagen gels were prepared by adding 0.5 ml 10× Eagle's MEM solution (Gibco) to 4 ml rat-tail type I collagen (First Link) in 0.1 M acetic acid, protein concentration 2.035 mg ml^−1^. The mixture was then neutralized with a combination of 5 M and 1 M sodium hydroxide, using the indicator colour changes from yellow to cirrus pink to specify neutralization [21]. While still in liquid form, the neutralized gel preparation was mixed with the cell suspension (2 × 10^6^ cells in 0.5 ml medium), and the resultant 5 ml gel transferred into a mould (2.2 × 3.3 × 1 cm^3^) to set for 30 min at room temperature in a laminar flow hood. Once set, the gels were compacted by a combination of compression and blotting using layers of mesh and paper sheets [1,11]. In short, a layer of nylon mesh (50 μm mesh thickness, purchased from John Lewis, London, UK) was placed on a triple layer of filter paper (Whatman paper, grade 1; 11 μm thickness, 185 mm diameter). The set collagen gel was placed on top of the nylon mesh, then covered with a second piece of nylon mesh and loaded with a 120 g flat steel for 5 min at room temperature. This produced a flat collagen sheet (approx. 80 μm thick) housed between the two pieces of nylon mesh. The top nylon mesh was peeled off, and the dense sheets of collagen were rolled using a sterile surgical blade and forceps to produce a tight spiral cylinder/rod measuring 3.5 mm in diameter and 21 mm long. Each spiral was constructed from 8 to 10 rolls denoting that each construct had 8 to 10 layers separating the outer from core segment (a schematic of the construct geometry is shown in figure 2—notably, alternate layers of collagen material are not in contact). The spirals were self-adhesive and stable, therefore unfurling was not an issue [11]. The final construct seeded two million cells at a density of 23.2 × 10^6^ HDFs ml^−1^ after a single plastic compression. The constructs were placed in culture well (six-well) containing 7 ml of complete culture medium and left to incubate at 37°C [1,11,31].

### 2.3. Cellular proliferation

Alamar blue is a redox indicator that changes colour and fluoresces in response to the chemical reduction of culture medium that results from cell proliferation and division [13,32]. Alamar blue (10× concentrate; Serotec) was diluted 1 : 10 with phenol red-free culture medium. The collagen constructs were unrolled using forceps and surgical blades at specific time points (day 0, 1, 3, 5, 7 and 10 incubation periods). These time points were selected to coincide with those for previous oxygen concentration gradient analysis conducted by the authors [1,31]. Constructs were cut (using stainless steel industrial cutting blades) into three equal segments measuring 20 × 21 mm representing the outer, middle and core of the three-dimensional construct. Segments were placed in culture wells (12-well) and incubated with 1 ml of the diluted Alamar blue solution at 37°C for 4 h. Three 100 μl Alamar blue solution samples were extracted from each segment and transferred into a 96-multiwell plate. Absorbance readings were taken at 510 and 590 nm using a microplate spectrophotometer (Labsystems Fluoroskan Ascent Machine). A standard curve was established to convert absorbance readings into cell numbers; cellular proliferation was deduced by analysing the percentage change in cell number compared with the day 1 time point in the outer region (calculated within each segment of each construct).

### 2.4. Cellular viability

Cell viability was quantified and assessed using a live/dead reduced biohazard viability/cytotoxity kit (Molecular Probes, L-3224), following the manufacturer's protocol. Live and dead cells were simultaneously identified with calcein AM (green) and ethidium homodimer (red) stains, respectively. The collagen constructs were unrolled using forceps and surgical blades as described above. The segments were placed into culture wells (12-well) and covered with 0.5 ml of the live/dead solution (20 μl of calcein AM/5 ml PBS added to 17 μl of ethidium homodimer/5 ml PBS, which equates to 2 μM calcein AM and 8 μM ethidium homodimer). The collagen matrices were incubated in live/dead solution at 37°C for 45 min before being mounted onto slides and viewed under an Olympus BX-UCB microscope. The green fluorescent nucleic acid and dead red stain were used to differentiate live and dead cells, respectively. Images were captured by the microscope and live/dead nuclei counted to ascertain percentage viability. Nine randomly selected areas within the outer, middle and core were analysed, resulting in 27 randomly selected areas being captured and counted within each construct. Data presented are live cells as a percentage of total cell number (live + dead).

### 2.5. Cellular migration

Cell migration was quantified using a cytoplasmic stain (CellTracker red (CMTPX), Invitrogen) conducted according to the manufacturer's protocol. The cells placed in the core of the spiralled collagen construct were labelled with a cytoplasmic stain and left to incubate for 10 days. At time intervals of day 0, 1, 3, 5 and 10, the spiralled collagen gel was cryopreserved and sectioned (5–8 μm thick) using the cryostat machine then mounted on a microscope slide as per protocol. The sections were viewed under an Olympus BX-UCB microscope and blue (none core cells) and red (originally core cells) fluorescent stains were used to determine the degree of migration from the core to the remainder of the construct. Images were captured by the microscope; ImageJ software was used to calculate the stain intensity at different spatial positions, and therefore analyse the degree of spread for the red stained cells (the stain density is assumed proportional to cell number, and is therefore a marker for cell density). Three repeats for each time point were conducted. Cumulative intensity data were normalized to calculate the probability of a cell residing within a fixed distance from the construct origin. Distances from the origin were measured both radially from the construct origin, and based on the distance moved round the spiral centreline (figure 2). This spiral distance was calculated through *L* = π *N* (*D*_0_ + *h* (*N* − 1)), where *D*_0_ = 80 μm is the inner diameter of the spiral, *N* = 9 is the number of turns and *h* = 80 μm is the thickness of the collagen sheet.

### 2.6. Statistical analysis

Data obtained using the protocols described above were analysed using IBM SPSS Statistics software v. 19. One-way and two-way ANOVA tests were used to detect significant differences between the datasets. A *p*-value of less than 0.01 (1% cut-off point) was deemed significant, and all data trends discussed correspond to this *p*-value.

## 3. Results

Plastic-compressed collagen gels exhibited strong spatial and temporal variations in their cellular distributions, quantified using proliferation, viability and migration results as described below. First of all, the spiral construct properties and oxygen monitoring results [1,31] are summarized, as oxygen gradients are hypothesized to drive observed changes in cellular density. Next, the results from the cellular proliferation, viability and migration studies are presented in turn.

Each spiralled collagen construct was cylindrical in shape, typically measuring 3.5 mm in diameter and 21 mm long. Each spiral was constructed from 8 to 10 rolls denoting that each construct had 8 to 10 layers separating the outer from core segment (a schematic of the construct geometry is shown in figure 2—notably, alternate layers of collagen material are not in contact). The final construct seeded two million cells at a density of 23.2 × 10^6^ HDFs ml^−1^ after a single plastic compression. Constructs were cut into three equal segments measuring 20 × 21 mm representing the outer, middle and core of the three-dimensional construct for future analysis.

Detailed results of oxygen dynamics have been reported previously [1,31]. Cellular constructs exhibited time-dependent oxygen depletion in their core and middle regions. Oxygen levels fell rapidly towards approximately steady-state or plateau values over the first hour, and the oxygen level at steady state varied according to the cell density (figure 1). For a starting cell density of 23.2 million cells ml^−1^, the oxygen levels after 20 h reached 22.3, 28.9 and 100.0 mmHg in the core, middle and outer regions, respectively. Over the 140 h experiment, the core levels recovered to approximately 43.2 mmHg; in the middle region, levels recovered to 61.1 mmHg at 101 h, and decreased by 12 mmHg over the next 37 h. Oxygen levels in the outer region showed a more moderate recovery to 116.0 mmHg at 103 h, and decreased to 100.0 mmHg again by 140 h. Once again, cell consumption was identified as the key factor influencing oxygen levels. Cell density determined the degree of oxygen depletion response, with seeded cell densities of 0, 5.8, 11.6 and 23.2 million cells ml^−1^ tested; however, spatial variations in cell density within an individual construct were not investigated.

### 3.1. Cellular viability

**Figure 3.**
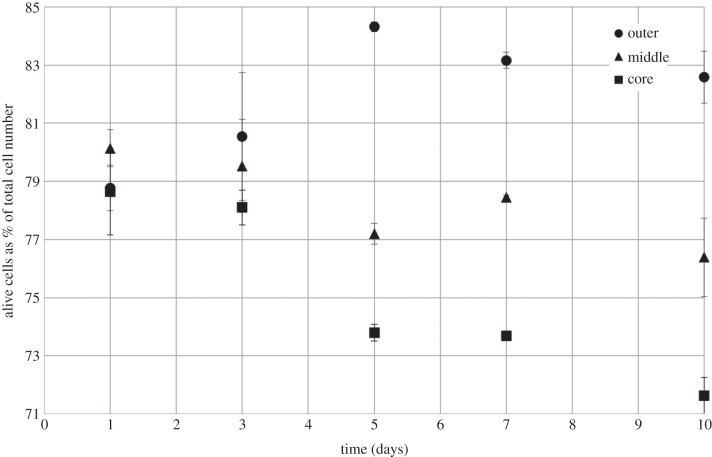
Percentage of live HDFs within the outer, middle and core regions of plastic-compressed collagen constructs. Data obtained using live/dead staining over a 10-day period. Increase in cellular viability in the outer region, and decrease in the middle and core regions, were statistically significant over the 10-day time period. No significant difference in cell viability in the outer segment over the first 3 days, significance only observed from day 5 onwards. Data are based on three independent experiments/repeats. Includes error bars for standard error.

**Figure 4.**
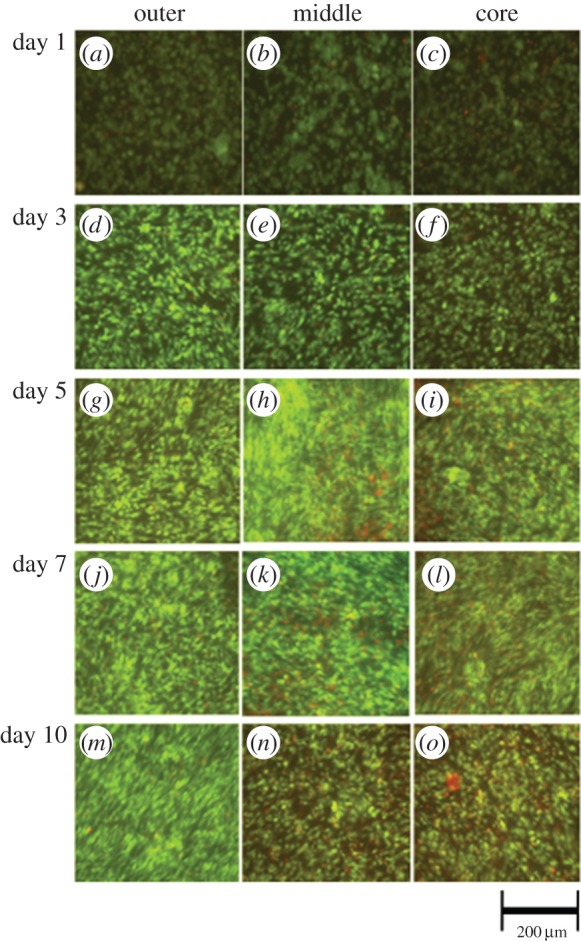
Confocal images of the outer, middle and core regions at days 1, 3, 5, 7, 10 after live/dead staining. Images show spatial and temporal variations in cellular viability and support the data presented in figure 3.

The spatial (outer, middle, core) and temporal (days 1, 3, 5, 7, 10) distribution of viable cells (as a % of total cell number) is depicted in figure 3, with supporting confocal images in figure 4. The cellular distribution of live cells changed over the 10 days within each of the defined sections. Small but statistically significant decreases in % viable cells were observed in both the middle and core segments over the 10-day experiment, falling from 80.1 to 76.4% and 78.6 to 71.6% in the middle and core regions, respectively. Indeed, the percentage of dead cells was highest in the core at day 10 (approx. 28.4% of cells were dead). By contrast, there was a significant increase in the percentage of live cells in the outer segment over the same 10-day time period, with cell viability increasing from 78.8% to a maximum of 84.3% at day 5, before falling slightly to 82.6% after 10 days. The difference in cellular viability between the outer and either middle or core regions was not statistically significant up to day 3, but differences measured at the 5, 7 and 10 day points were significant. The maximum difference between the outer and middle or core regions was observed at day 5, analogous to the cellular proliferation results (see below).

### 3.2. Cell proliferation

**Figure 5.**
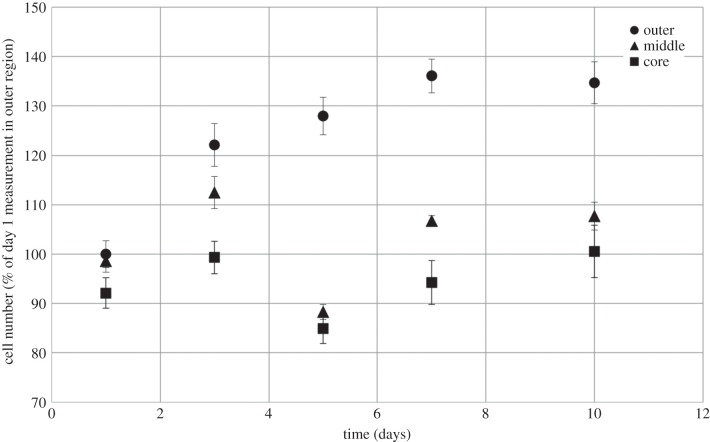
Proliferation of HDFs in plastic-compressed collagen constructs. Percentage change in cell number as a proportion of the day 1 time point in the outer region; graph shows measurements in each of the outer, middle and core regions of the construct. The increase in proliferative cell numbers across the outer, middle and core segment across the 10-day period measured was statistically significant. Data are based on three independent experiments/repeats. Includes error bars for standard error.

Cell proliferation was highest within the outer segment of HDF-laden plastic-compressed collagen gels compared with the middle and core segments, as shown by a statistically significant increase in the percentage of cells detected by the Alamar blue-specific fluorescence (figure 5). There was a 36.1% increase in the number of cells within the outer segment over the first 7 days, with cell number (compared with the day 1 outer region value) then remaining approximately constant at 134.7% on day 10. Although there is no statistically significant change in cellular proliferation across the middle and core segments up to day 3, significant proliferation was measured in these segments from day 5 to 10 (19.5% and 15.67% increases for the middle and core, respectively).

Indeed, HDFs seeded in the outer segment of the collagen construct for days 3, 5, 7 and 10 showed significantly higher proliferation compared with those seeded in the middle or the core of the same construct. The difference in cell number between the outer and the core was greatest at day 5 (43.1%), with a 22.8, 41.9 and 34.2% difference, respectively, for days 3, 7 and 10 (figure 5). Similarly, a 39.7% difference between the outer and middle segments was detected on day 5, compared with 9.6, 29.3 and 27.1% on days 3, 7 and 10, respectively. Crucially, there was no significant difference in proliferation across the segments at day 1.

### 3.3. Cell migration

The cohort of cells originally placed in the core of the construct was stained and subsequent intensity analysis used to evaluate their mean position over the 10-day period. Specifically, the distance between the construct origin and the mean cellular position (within which 50% of cells resided) was calculated, using both radial and spiral descriptions (see figure 2 for a description of the two coordinate systems, and the relationship between the two). The direction of movement under chemotaxis (if it occurs) is not known *a priori*; spiral movement would involve cell migration around the axis of the spiral geometry within the collagen gel matrix (note that the collagen layers are not in contact). By comparison, cell migration in a radial direction would require cells to alternatively move out of the collagen matrix into fluid-filled space, then back into the collagen matrix, that is, across each collagen layer in turn (figure 2).

**Figure 6.**
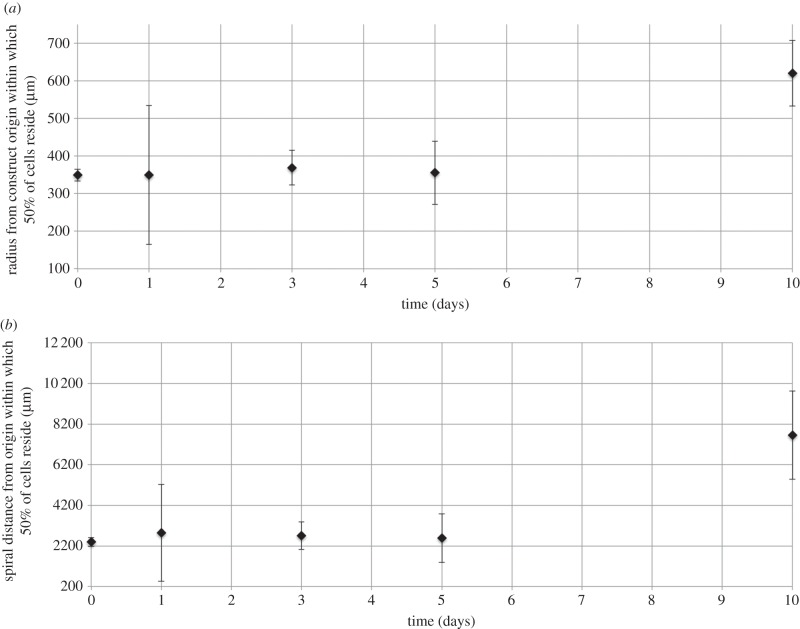
Distance from the origin within which 50% of cells that originated in the core region reside, measured either radially (*a*) or along the spiral centreline (*b*) from the construct origin. Cells placed in the core of the spiralled construct were labelled with nucleic acid stain, and stain intensity (which is proportional to cell density) tracked at subsequent time points. Cumulative intensity data were normalized to calculate the probability of a cell residing within a fixed distance from the construct origin, measured either radially or round the spiral centreline (figure 2). Data are based on three to five independent experiments/repeats. Includes error bars for standard error.

As shown in figure 6, there was no statistically significant change in the mean cellular position up to 5 days; however, from day 5 to 10, the mean position of cells originating in the core of the construct did move outwards, indicating chemotactic behaviour. These observations are both true, independently of the coordinate choice for distance measurement. The mean cellular position begins on day 0 at a radial distance of 349 μm from the construct origin, with this distance being maintained at 355 μm at the day 5-day time point; however, the radial distance from the origin increases by 74.4% to 620 μm between days 5 and 10 (figure 6*a*). Analogously, on day 0, the spiral distance from the origin is 2396 μm; this is maintained at 2582 μm at day 5, but increases by 197% to 7656 μm by day 10 (figure 6*b*). Notably, the mean position still lies within the core region after 10 days (reaching the core/middle region interface corresponds to moving 1006 μm radially and 19 869 μm in the spiral direction, from the construct origin). The spread of the cellular distribution does not vary in time (data not shown), indicating that cells move as a cohort. In addition, stained cells remain within the collagen matrix layers (data not shown), indicating that cells migrate by moving through the collagen matrix (rather than on its surface, or within the fluid layers).

**Figure 7.**
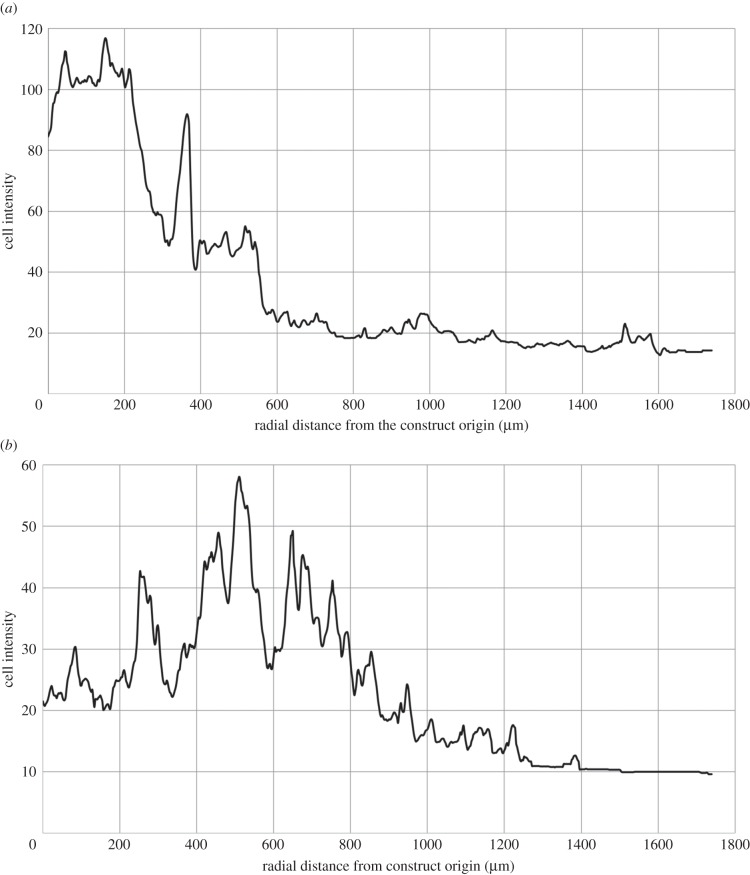
Change in cell intensity distribution at day 5 (*a*) and day 10 (*b*) for fluorescently labelled cells originating in the construct core (cell intensity is proportional to cell density). Net migration from a mean radial position of 355–620 μm is apparent over the 5-day time period. A periodic pattern of peaks and troughs is apparent, and has period approximately equal to the thickness of the collagen sheet (80 μm).

The speed of migration over the day 5–10 time period corresponds to 392 μm per day radially and 1015 μm per day round the spiral. Figure 7*a*,*b* shows the change in the intensity plots for day 5 compared with day 10, and the net migration from a mean radial position of 355–620 μm is apparent. The periodic pattern of peaks and troughs, with period approximately equal to the thickness of a collagen sheet (80 μm), is indicative of migration around the axis of the spiral geometry within the collagen gel matrix. This pattern was accentuated by day 10, and is discussed further in §4.

## 4. Discussion and conclusion

A current challenge in the field of tissue engineering and three-dimensional culture remains the lack of quantitative information linking nutrient delivery and cellular distribution. This is despite a common concern in the literature for the ability of ‘deeper-lying’ cells to receive adequate oxygen to support physiological function. *In vivo*, oxygen is supplied to tissue by diffusion from the nearest blood vessel; however, in three-dimensional *in vitro* static culture systems, cells rely purely on diffusion from the construct margins. As a consequence, the oxygen level at a defined distance from the oxygen source depends critically on the balance of oxygen diffusion and cellular metabolism; the latter is both cell-type-specific and a function of the underlying cell density. Indeed, the picture is further complicated by the fact that cells respond to their local oxygen environment through proliferation, death and chemotaxis. The rate of cellular proliferation depends critically on the underlying availability of oxygen, whereas death (through apoptosis or necrosis) may occur in oxygen-deprived zones. For cell types with a migratory potential, chemotaxis along a defined oxygen gradient will also result in spatially resolved gradients in cellular density. In order to culture three-dimensional tissues with physiological function, it is of fundamental importance that the basic features of the *in vivo* environment are mimicked and this requires a detailed understanding of the interplay between nutrient status and cellular behaviours.

In spite of the above, there remain few simple three-dimensional models that enable the interplay between oxygenation and cell fate to be quantified. Recent developments in plastic compression and layer assembly technologies for tissue fabrication have produced three-dimensional cultured constructs with definable, near-native properties for which oxygenation has been measured and spatially resolved. Previous studies [1,31] have measured the spatial oxygen gradient established in such constructs through the balance between diffusion and cellular metabolism; the present study expands this to quantify the resulting gradients in cell density, as well as the mode of chemotaxis of HDFs under a controlled oxygen gradient.

The defining features of the three-dimensional collagen-based culture system under investigation are analogous to those at play for *in vivo* tissues: (i) cell density, which varies both spatially and in time, (ii) matrix permeability to the limiting diffusing component (oxygen is considered to be limiting nutrient in the present study), (iii) metabolic demand of the cell population (e.g. chondrocyte, cardiomyocytes, dermal fibroblast), and (iv) distance for diffusion from the oxygen source. The plastic-compressed system enables control of each of these features. The matrix density and construct size are determined by the initial collagen content, extent of compression and fabrication process (indeed, the collagen matrix has a nanofibrillar lattice of around 88% water, making it highly permeable to oxygen). The seeded cell density is determined by the initial inoculum, and could be spatially controlled if this gave rise to a cultured cell distribution that better mimicked the *in vivo* scenario. Finally, the cell type choice and cellular density determine the total metabolic demand of the cell population (which, again, is spatially varying).

Previous studies have quantified the oxygen gradients established in such systems under culture of HDFs or HBMSCs at defined and uniform seeded cell densities [1,31]. In all cases, oxygen levels fell rapidly towards approximate equilibration in the first hour of the experiment; the rate of fall of oxygen levels and equilibrium consumption of oxygen was entirely predictive, and dependent on cell density. Cell viability at the construct core was maintained above 70% in all cases, and no zone of the construct ever became pathologically hypoxic. For HDFs seeded at a density of 23.2 million cells ml^−1^, an oxygen partial pressure gradient of 44.5 mmHg mm^−1^ was established radially from the outer surface to the core of the construct after 20 h (figure 1). Whereas oxygen levels in the construct core then gradually increased by approximately 94% over the experimental time scale, those in the middle and outer regions recovered to local maxima after around 100 h, before decreasing marginally by the end of the experiment. The final oxygen partial pressures in the middle and outer regions were 13.7% and 131.5% higher than that in the core, respectively.

In this study, changes in cell density linked to this temporally varying oxygen gradient field were resolved spatially. This was achieved by unrolling cultured spirals at defined time points, and analysing cellular content and behaviours in the outer, middle and core regions of the underlying collagen sheet. Cellular proliferation and chemotaxis were explored through metabolic activity stains, and also by tracking the progression of fluorescently labelled cells seeded in the construct core. The combined impact of proliferation, chemotaxis and death was also explored through cellular viability studies.

Cell proliferation and viability analysis elucidated the correlation of cellular proliferation and viability with oxygen tension. Cells throughout the construct stayed viable over the 10-day culture period. In general, cellular viability decreased with corresponding oxygen partial pressure; for example, at day 10, 82.6% of cells in the oxygen-rich outer segment remained viable, whereas this was 7.5 and 13.3% lower in the middle and core, respectively. These data correlate well with information generated from previous studies [1,11,31,33]. Throughout the 10-day experimental period, the proportion of viable cells in the outer segment increased, whereas the number of viable cells in the core decreased. This is consistent with trends in proliferative cell number obtained using Alamar blue staining (figure 5), where cellular proliferation rates were highest in the outer segment. The results of the Alamar blue also show that whereas the main site of cellular proliferation was the outer segment, significant division occurred in the later stages within the core as well. Indeed, there was a net increase in proliferative cell number in the middle and the core over the experiment. In the outer segment, cellular viability increased to a maximum at day 5 and then plateaued. This is indicative of an increase in proliferation and division in the outer segment diluting the number of dead cells within that construct, and is consistent with previous studies [13].

Cellular proliferation was tightly correlated with spatial position and thus the underlying oxygen field. Rate of proliferation was highest in the high-oxygen outer region of the construct, where cells with metabolic activity increased by 36.1% over a 7-day period. The increase in proliferative number was fastest over the first 5 days (an increase of 28.0%); the higher cell numbers during the day 5–10 time period resulted in a higher net metabolic demand by the cell population, and is the likely cause for the gradual decrease in the oxygen levels in the outer region from 100 h onwards. Indeed, the reduction in rate of proliferation over days 5–10 is indicative of a maximum tolerance of cell density, perhaps as a consequence of contact inhibition [13].

Within the lower oxygen environment of the construct middle or core regions, rates of proliferation were lower by comparison. Over the first 3 days, cell number increased in both regions; however, these dropped by day 5, and increased again by day 10. Such oscillatory behaviour is consistent with proliferation patterns in previous studies [13] and is hypothesized to indicate that the cell population is hovering about its saturation point for a specific oxygen concentration. Indeed, previous studies have shown that a single compressed construct containing HDFs should not exceed 1.8 million cells [33], which is consistent with saturation for the current model. Further, the culture medium was replenished every 3 days; this introduces ‘fresh’ oxygen into the system that may induce oscillations in cell numbers in the middle/core of the scaffold (but not in the outer region where oxygen levels, cell density and net metabolism are all higher).

The delicate balance between cell density and oxygen tension is further borne out by the cellular chemotaxis analysis. The cohort of cells seeded in the construct core remained in their original location for the first 5 days of the experiment; however, chemotaxis was observed between days 5 and 10 with a net migration from the construct core towards the middle region (i.e. from a lower to a higher oxygen environment). This behaviour would be anticipated to continue over a longer experimental time scale; although the mean cell position did not leave the core region over the 10-day period, this could easily be achieved over a longer experiment. Although this chemotactic migration of cells from the core region outwards will have contributed to the increase in cell numbers with metabolic potential in the outer region, these migration data indicate that chemotaxis mainly occurred within the core region over the experimental time scale. Therefore, it seems probable that the increase in number of cells with metabolic activity arose through proliferation of cells that originated in the outer region.

Plots of the cell intensity as a function of radial distance from the construct origin (figure 7) indicate a periodic pattern of peaks and troughs that coincides with the collagen sheet thickness (indeed analysis of histological sections indicated that stained migratory cells remained with the collagen matrix—data not shown). This indicates that cells remained within the collagen matrix sheets (which were not, in general, in contact), and therefore migrated along the spiral direction of the construct (we note that regions where alternate collagen layers are in contact will thus provide a ‘short-circuit’ for migration, although analysis of sections through the construct did not indicate this happened frequently enough to skew the analysis here). This is of significant interest—there is an underlying competition between the direction of the most acute oxygen gradient (which is radial) and the cost of migrating radially through alternate layers of collagen matrix and fluid compared with a matrix-only route in the spiral direction. For example, at day 5, this oxygen gradient is 35.1 mmHg mm^−1^ from the outer to the core radially versus 1.03 mmHg mm^−1^ spirally. This analysis indicates that the spiral route ‘wins’—cells appeared to migrate from lower to higher oxygen regions along the spiral from day 5 to 10, with a mean speed of 1015 μm day^−1^. To the best of our knowledge, there are no other studies which quantify chemotactic migration rates for this cell type.

Cell migration along the spiral was only observed from day 5 onwards (figure 6), where a corresponding drop in both the percentage live/dead and proliferation occurred in the core of the constructs (figures 3 and 5). Therefore, until day 5, or day 3 at the earliest, cell viability within the core of three-dimensional constructs was not adversely affected by the low oxygen environment. Cell migration from day 5 onwards appears to correspond to an increase in cell death and a drop in cell proliferation, so cells are migrating in response to a threshold level of oxygen, which is not sustaining cell viability. This cell migration was modest given that the mean cell position remained within the core; however, it was efficient in rebalancing the system, as evidenced by the recovery of the oxygen partial pressure and proliferative cell density in the core by the end of the experiment. The system then maintained a constant cycling around a stable maximum cell density, driven by the interplay between oxygen tension, cellular proliferation, death and chemotaxis.

In summary, we have established a basic model for the study of the interplay between cell density and oxygen consumption by HDFs in three-dimensional collagen matrices. The tight correlation between tissue oxygenation and cellular proliferation, viability and chemotaxis was demonstrated and quantified. Preferential cellular proliferation and viability occurred in the high-oxygen zones in the outer region of the constructs; in addition, cellular chemotaxis resulted in migration of cells around the construct spiral from lower to higher oxygen regions. The study provides insight into the likely natural mechanism for adapting cell number and activity to a prevailing oxygen regime, based on cellular proliferation, death and migration, giving rise to a constant cycling in cell density around a stable base state. This raises the possibility of engineering tissues with defined and controllable gradients in both cell density and oxygenation, with a view to mimicking *in vivo* effects. In addition, these quantitative data provide information to guide vascularization studies, for example by guiding the location and extent of printed channels required to maintain tissue function.
